# Thyroid hormones and ovarian reserve: a comprehensive study of women seeking infertility care

**DOI:** 10.1186/s12905-023-02725-1

**Published:** 2023-11-04

**Authors:** Muge Halici, Mustafa Ege Seker, Irem Yagmur Gebedek, Merve Nida Gokbak, Abdurrahman Furkan Cetisli, Ahmet Berkan Ciftci, Emine Konac, Sule Yildirim Kopuk, Bulent Tiras, Yigit Cakiroglu

**Affiliations:** 1 School of Medicine, Acibadem Mehmet Ali Aydinlar University, Atasehir, Istanbul, 34752 Turkey; 2 Assisted Reproductive Technologies Unit, Acibadem Maslak Hospital, Sariyer, Istanbul, 34398 Turkey

**Keywords:** AFC, AMH, FSH, Infertility, Ovarian reserve, TSH

## Abstract

**Background:**

Ovarian reserve is the number of oocytes remaining in the ovary and is one of the most important aspects of a woman’s reproductive potential. Research on the association between thyroid dysfunction and ovarian reserve has yielded controversial results. In our study, we aimed to investigate the relationship between thyroid-stimulating hormone (TSH) levels and ovarian reserve markers.

**Methods:**

From 1443 women seeking infertility care, the data of 1396 women aged between 20–45 years old who had a body mass index between 18–30 kg/m^2^ were recruited for this retrospective study. The anti-Müllerian hormone (AMH) and TSH relationship was analyzed with generalized linear and polynomial regression.

**Results:**

Median age, follicle-stimulating hormone (FSH), AMH, and TSH levels were 36.79 years, 9.55 IU/L, 3.57 pmol/L, and 1.80 mIU/L, respectively. Differences between TSH groups were statistically significant in terms of AMH level, antral follicle count (AFC), and age (*p* = 0.007 and *p* = 0.038, respectively). A generalized linear regression model could not explain age-matched TSH levels concerning AMH levels (*p* > 0.05). TSH levels were utilized in polynomial regression models of AMH, and the 2^nd^ degree was found to have the best fit. The inflection point of the model was 2.88 mIU/L.

**Conclusions:**

Our study shows a correlation between TSH and AMH values in a population of infertile women. Our results are as follows: a TSH value of 2.88 mIU/L yields the highest AMH result. It was also found that AMH and AFC were positively correlated, while AMH and FSH were negatively correlated.

**Supplementary Information:**

The online version contains supplementary material available at 10.1186/s12905-023-02725-1.

## Background

Infertility is defined as a couple’s inability to conceive after one year (for women younger than 35) or six months (for women older than 35) of regular unprotected sexual intercourse [1]. It can be due to female factors, male factors, both female and male factors, or unexplained infertility [2]. Age, acute or chronic disorders, genetics, environmental exposures, lifestyle factors, infectious diseases, and specific reproductive disorders can affect either sex attempting to become pregnant [1].

Ovarian reserve is the number of oocytes remaining in the ovary and is one of the most important aspects of a woman’s reproductive potential [3]. Follicle-stimulating hormone (FSH) and anti-Müllerian hormone (AMH) measurements are biochemical tests that predict ovarian reserve. Since FSH secretion is inhibited by ovarian hormones, the elevation of FSH indicates poor production of ovarian hormones and, thus, a diminished ovarian reserve. Conversely, AMH is produced by granulosa cells of small, large preantral and small antral follicles. Therefore, a decline in serum AMH levels indicates a reduction in ovarian reserve [4, 5]. Another method for ovarian reserve testing is ultrasound imaging of the ovaries to detect antral follicle count (AFC). AFC is the summation of 2–10 mm follicles, which are defined as antral follicles by most studies [6]. Average AFC differs according to age. Nevertheless, an AFC of 5 is considered low and indicates a decrease in ovarian reserve [7].

The question of whether thyroid dysfunction is associated with ovarian reserve has been studied by many researchers. This question was born out of the fact that many components of female reproductive health are associated with thyroid function. For example, menstrual disturbances can be seen in thyroid dysfunction and overt hyperthyroidism has been linked to some obstetrical complications, such as spontaneous abortion and preterm delivery [8]. However, research on the relationship between thyroid function and ovarian reserve has yielded controversial results.

A correlation between thyroid-stimulating hormone (TSH) and ovarian reserve in infertile populations was demonstrated by some studies [9–11]. However, investigations revealed that TSH levels and ovarian reserve were not correlated in a study performed with women without prior known thyroid disease or infertility [12]. A Belgian study of 5000 women found no relationship between TSH levels and low ovarian reserve when infertility diagnosis was considered and not considered [13]. As such, a retrospective cohort study conducted with 256 women followed by a systematic review and meta-analysis showed no correlation between TSH levels and In Vitro Fertilization (IVF) treatment results in euthyroid women [14]. On the other hand, a study claimed that the success of IVF treatment in terms of the number of oocytes retrieved and pregnancy rate is inversely related to hypothyroidism without autoimmune disease and Hashimoto’s thyroiditis, even when the laboratory parameters of the patients were corrected with proper treatment [15].

As studies could not agree on this topic, new studies are still needed to examine the relationship between thyroid function and ovarian reserve.

Our study aimed to investigate this controversial topic by examining the association between TSH groups and ovarian reserve markers such as AMH, FSH, and AFC.

## Methods

### Population

For this cross-sectional study, patients between 2015 to 2021 from the Acibadem Maslak IVF Unit were recruited. Women with a history of infertility for over a year, aged between 20 and 45 years old, and with a body mass index (BMI) between 18 and 30 kg/m2 were included in the study population. Women who had any prior thyroid treatment (medical, radioactive iodine, or surgical) or had been diagnosed with Graves' disease, Hashimoto's thyroiditis, papillary thyroid carcinoma, hypophyseal adenoma, and hyperprolactinemia were excluded. We also excluded women who had any uterine lesions or ovarian cysts. Of the remaining 1443 patients, 47 were excluded due to missing values. The analysis was carried out on 1396 patients. Post-hoc study power was calculated as 0.983 with a small (0.1) effect size and error probability of 0.05.

**Fig. 1:**
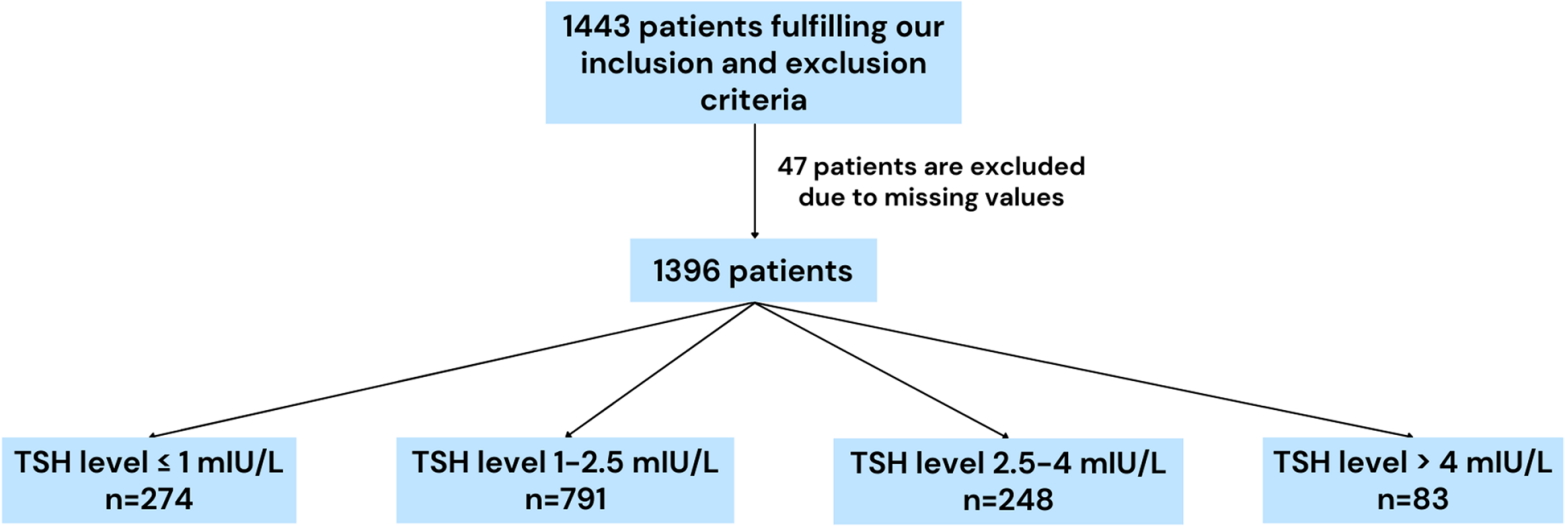
Study flowchart showing the number of included patients and the TSH groups

As part of routine pre-IVF evaluation, AMH, TSH, and FSH assessments were made in the blood serum, and AFC was determined using transvaginal ultrasonography by the same proficient physician (YC) on days 2–4 within the onset of the menstrual cycle. The electrochemiluminescence immunoassay “ECLIA” method was utilized to measure AMH with Roche Cobas E411 (measuring range: 0.07–164 pmol/L; limit of blank = 0.049 pmol/L, limit of detection = 0.07 pmol/L, limit of quantification = 0.214 pmol/L) while TSH (measuring range: 0.008–150 mIU/L; limit of blank ≤ 0.004 mIU/L, limit of detection ≤ 0.008 mIU/L, limit of quantification ≤ 0.008 mIU/L) and FSH (measuring range: 0.3–200 IU/L; limit of blank ≤ 0.3 IU/L, limit of detection ≤ 0.6 IU/L, limit of quantification ≤ 0.6 IU/L) was measured using the chemiluminescence CLIA method using Siemens Atellica IM. For the study, these values and additional information, such as age, infertility type, and menstrual history, were obtained from each patient’s electronic medical records. Menstrual history was divided based on The International Federation of Gynecology and Obstetrics (FIGO) consensus. Regular menses were described as menses that have a frequency of 24 to 38 days and last shorter than eight days. On the other hand, irregular menses were the menses that did not cover these conditions [16]. Participants were placed into one of the four groups according to their TSH level. To determine these four groups, we divided the TSH range as ≤ 1 mIU/L, 1–2.5 mIU/L, 2.5–4 mIU/L, and > 4 mIU/L. The limits for the TSH range were based on literature research [17, 18]. The study flowchart stating the number of included patients and the TSH groups are shown in Fig. 1. Statistical analyses were performed between TSH groups and other parameters to determine whether there is an association between thyroid hormones and ovarian reserves.

This study was approved by the Acibadem Health Group Ethical board (ATADEK) (2021–07/26). As Turkish laws state that if the anonymity of the patients’ identities is protected, a retrospective review of medical records does not require informed consent to be taken from the patients, ATADEK waived the need for informed consent.

### Statistical analysis

All data were stored in SPSS (Statistical Product and Service Solutions) 22 statistical software [19]. All analyses were executed in R Studio [20] with R statistical software [21]. In the performed tests, a confidence level of 95% was considered significant (*p* < 0.05).

First, a descriptive study of the samples was carried out. The skewness and kurtosis of the samples were analysed. We carried out The Shapiro–Wilk test out to assess the normality of data. Continuous parameters were found not to be normally distributed; thus, they are described with median (interquartile). Qualitative parameters are described by absolute frequencies.

**Fig. 2:**
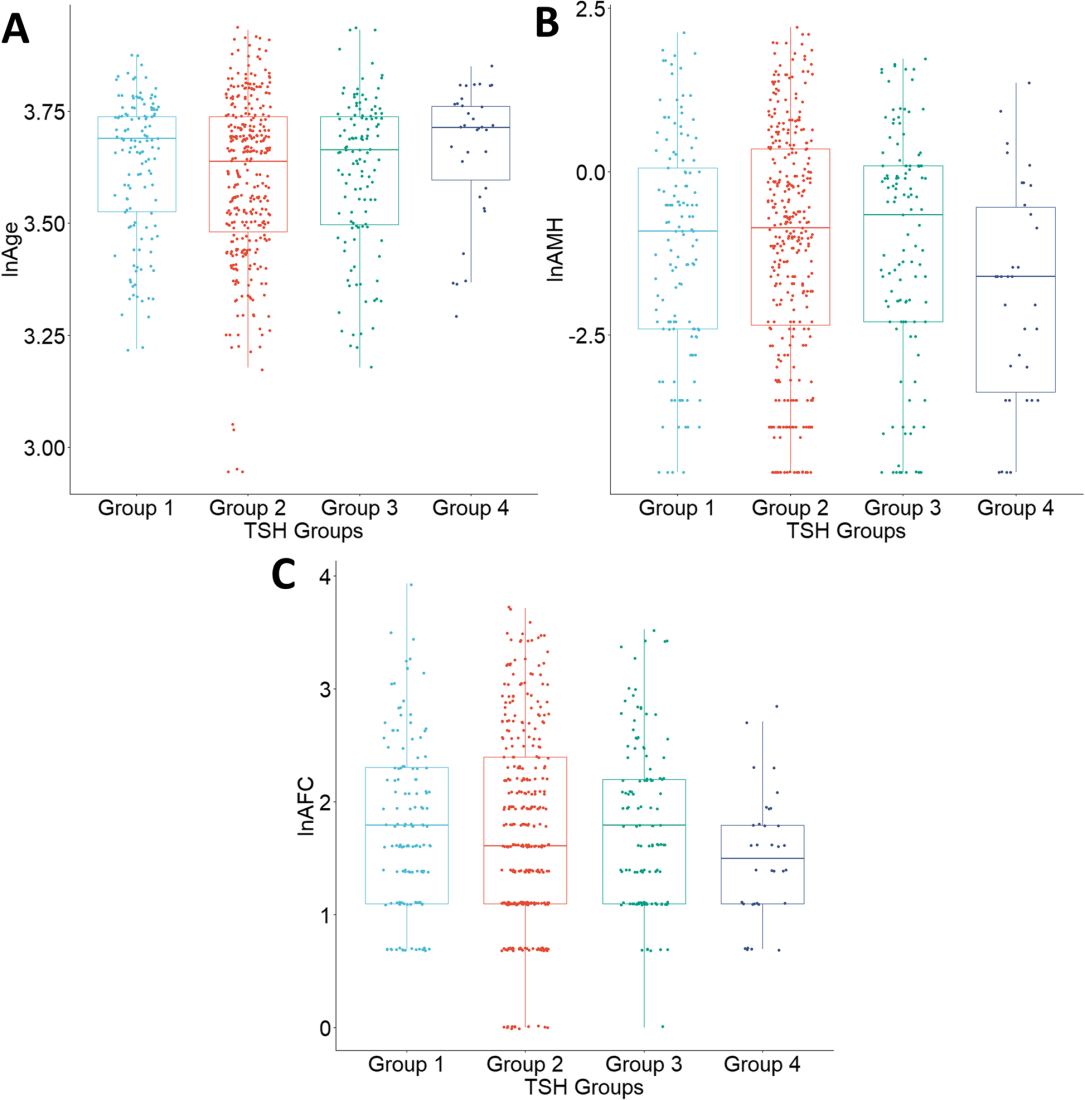
Graphs for age, AMH, and AFC in group comparisons. Abbreviations: lnAge: Logarithmic transformation of age parameter, lnAMH: Logarithmic transformation of anti-Müllerian hormone parameter, lnAFC: Logarithmic transformation of antral follicle count parameter

Group comparisons for continuous parameters were made by the Kruskal–Wallis test and post hoc by the Dunn’s test. The categorical variables were analyzed by the chi-2 test. The distributions of the groups are further demonstrated in Fig. 2. Due to the nonparametric nature of the parameters, logarithmic transformation was utilized. In the AFC variable, one unit was added to all dataset to allow the utilization of values of zero [22–24].

The age parameter was significantly different between TSH groups; thus, the toolkit for weighting and analysis of nonequivalent groups (twang) was utilized [25] to minimize the effect of age. Herby generalized linear regression analysis was carried out to check the relationship between age-matched TSH groups and AMH levels.

Linear and polynomial linear regression analyses were carried out to check for a relationship between TSH levels and AMH levels (1^st^, 2^nd^, 3^rd^, 4^th^, and 10^th^ degrees were utilized).

Correlations between AMH-AFC and AMH-FSH were analyzed with Spearman correlation to make future assumptions about AFC and FSH.

## Results

From 1443 women seeking infertility care, we used the data of 1396 women. The median age and FSH, AMH, and TSH levels were 36.79 years, 9.55 IU/L, 3.57 pmol/L, and 1.80 mIU/L, respectively.

The parameters of the participants are summarized in Table 1. Differences between the groups were not statistically significant in terms of gravida and FSH levels (*p* > 0.05). Group differences were statistically substantial regarding AMH level, AFC, and age (*p* = 0.007, *p* = 0.038, and *p* < 0.001, respectively). Differences in AMH level medians between TSH groups 1 and 2, 1 and 3, 2 and 4, and 3 and 4 were found to be statistically significant (AMH median levels of the TSH groups were 3 pmol/L, 3.71 pmol/L, 5.5 pmol/L, and 1.71 pmol/L, respectively). Differences in AFC medians between TSH groups 1 and 3, and, 3 and 4 were found to be statistically significant (*p* = 0.011 and *p* = 0.016, respectively) (Table 1).

**Table 1:** Baseline characteristics of patients

	TSH Group 1 (TSH level ≤ 1 mIU/L) (*n* = 274)	TSH Group 2 (TSH level 1–2.5 mIU/L) (*n* = 791)	TSH Group 3 (TSH level 2.5–4 mIU/L) (*n* = 248)	TSH Group 4 (TSH level > 4 mIU/L) (*n* = 83)
Age (years)	39 (10)	36 (10)	37 (9)	40 (8)
Infertility type				
-Primary	167 (60%)	476 (60%)	151 (60%)	56 (67%)
-Secondary	107 (40%)	315 (40%)	97 (40%)	27 (33%)
Gravida	0 (1)	0 (1)	0 (1)	0 (1)
Menstrual cycle				
-Regular	210 (77%)	598 (76%)	190 (77%)	59 (71%)
-Irregular	64 (23%)	193 (24%)	58 (23%)	24 (29%)
FSH (IU/L)	9.2 (8.83)	10 (12.1)	9.44 (10.85)	10.4 (11.39)
AMH (pmol/L)	3 (7.86)	3.71 (12.64)	5.5 (11.79)	1.71 (6.5)
AFC	5 (7)	5 (10)	6 (10)	4 (4)

*Abbreviations*: *TSH* Thyroid-stimulating hormone, *FSH* Follicle-stimulating hormone, *AMH* Anti-Müllerian hormone, *AFC* Antral follicle count. The values shown in the table are given as median (interquartile) for continuous values and absolute frequencies for qualitative parameters

The *p*-values were derived from the Kruskal–Wallis test and post hoc by the Dunn’s test for constant parameters and the chi-2 test for qualitative parameters. Age differences between the TSH groups 1 and 2, 1 and 3, 2 and 4, 3 and 4 are statistically significant. AMH differences between the TSH groups 1 and 2, 1 and 3, 2 and 4, 3 and 4 are statistically significant. AFC differences between TSH groups 1 and 3, 3 and 4 are statistically significant

The propensity scores of each TSH group were calculated, and the effects of age were minimized. A weighted new dataset was used in the generalized linear regression model to predict AMH levels. AMH levels could not be explained by the model with age-matched TSH groups, which is summarized in Table 2. Due to restrictions of twang, polynomial regression models could not be utilized. Another linear regression model assessing the effects of TSH levels, infertility type, menstrual status, and age, is summarized in Table 3.

**Table 2:** Generalized linear regression model with age-matched TSH group data for AMH prediction

Variables	Estimate	Standard Error	t value	*p* value
TSH Group 1	1.16	0.11	10.33	< 0.001
TSH Group 2	0.14	0.13	1.05	0.295
TSH Group 3	0.10	0.16	0.66	0.511
TSH Group 4	0.02	0.29	0.07	0.948

*Abbreviations: TSH* Thyroid-stimulating hormone, *TSH Group 1* TSH level ≤ 1 mIU/L, *TSH Group 2* TSH level 1–2.5 mIU/L, *TSH Group 3* TSH level 2.5–4 mIU/L, *TSH Group 4* TSH level > 4 mIU/L

**Table 3:** Linear regression model for AMH prediction

Variables	Estimate	Standard Error	t value	*p* value
Intercept	5.22	0.26	19.62	< 0.001
TSH	0.017	0.03	0.48	0.625
Age	-0.11	0.006	-16.09	< 0.001
Infertility type	0.39	0.08	4.47	< 0.001
Menstrual regularity	-0.28	0.1	-2.83	0.004

*Abbreviations*: *TSH* Thyroid-stimulating hormone

TSH levels were utilized in polynomial regression models of AMH to different degrees. The 2^nd^ degree was found to have the best fit (Table 4). Other models can be found in Appendix Table (see Additional file 1). The TSH level inflection point of the model was 2.88 mIU/L.

**Table 4:** Linear regression models and polynomial regression models with TSH data for AMH prediction

Variables	Estimate	Standard Error	t value	*p* value
2^nd^ Degree Polynomial Regression Model				
Intercept	0.99	0.13	7.33	< 0.001
1^st^ Slope	0.26	0.09	2.66	0.008
2^nd^ Slope	-0.05	0.02	-3.03	0.003

AMH levels and AFC were positively correlated (rho: 0.809, *p*=<0.001). AMH and FSH levels were negatively correlated (rho: -0.654, *p*=<0.001).

## Discussion

Our study investigated whether there was an association between TSH values and ovarian reserve markers such as AMH, FSH, and AFC in a population of 1443 infertile women. It was shown in our analyses that the association between TSH and AMH can be best explained in a 2^nd^ degree polynomial regression model, and the inflection point of the model was 2.88 mIU/L, which means that the highest AMH result was yielded by a TSH value of 2.88 mIU/L.

With 225 infertile women, a retrospective study was performed by Weghofer et al. [9]. Only women within the normal TSH range (0,4–4,5 mIU/L) were included in this study. Women with a TSH level of < 3 mIU/L were found to have significantly higher AMH levels, even when adjusted for age. As it was found in our analyses that the optimal level of TSH is between 2 and 3 mIU/L, our study and this study show similarity in terms of results. Differently, it has been shown in our study that there is a more specific optimal TSH level, which is 2.88 mIU/L. In addition, our sample size was significantly larger than this study, and a specific TSH range was not set as an inclusion criterion. Thus, a more extended TSH range could be analyzed with our study.

Matching 67 infertile women with 27 fertile women according to BMI and age, a study was conducted by Kuroda et al. [10]. In the infertile population, it was shown that TSH levels and age have an effect on AMH levels both in prematch and postmatch results. However, in the fertile population, when assessed postmatch, AMH was not associated with either TSH or age. Our study population only consisted of women who had infertility. The age variable was adjusted in the TSH groups in our study; on the other hand, this was done for AMH in the stated study. An association between TSH and AMH was found in both our study and the stated study. However, in the stated study, they were inversely associated.

A total of 775 women were divided into four age-specific AMH quartiles in a study performed by Bahri et al. [12]. A significant difference between study groups in terms of thyroid dysfunction could not be found. This study used a different statistical approach from ours. Our study found that both high TSH and low TSH levels result in a low AMH level; thus, the association between TSH and AMH is not linear. Since this means that the low AMH group should consist of both high and low TSH levels, it is plausible that a difference between AMH groups could not be found in the stated study due to categorization being done according to AMH values. As our statistical approaches are different, their results do not contradict ours. In contrast, it strengthens them.

A total of 4894 patients, both fertile and infertile, were investigated in a cross-sectional retrospective study conducted by Polyzos et al. [13]. Patients were categorized and analyzed according to their AMH levels; however, in our study, these were done according to TSH levels. Polyzos et al. found that TSH levels and overt or subclinical hypothyroidism diagnoses did not demonstrate any differences between these different AMH groups. A subgroup analysis was also performed to assess thyroid function and ovarian reserve in infertile participants. This analysis did not show any significant difference between the groups regarding TSH and diagnoses of overt or subclinical hypothyroidism. Since the categorization was performed according to AMH values, the findings of this study do not contradict our findings.

No association of TSH with AFC and FSH levels was reported in a study performed by Korevaar et al. [26]. Our study focused on the association of TSH with FSH, AFC, and additionally AMH. In contrast to the stated study, a 2^nd^ degree polynomial association between TSH and AMH levels was found within our study.

It is worth mentioning that chronic anovulation is one of the most important causes of female infertility. The possible treatment of this condition was reported by some natraceutical supplementations such as D-chiro-inositol and Myo-inositol [27, 28]. It is also crucial to analyze neonatal outcomes and long-term follow-up of children born from assisted reproductive technologies (ART). Even though the research techniques have developed over the years, studies show chromosomal abnormalities of embryos derived from frozen oocytes and various outcomes based on different freezing techniques used in ART [29]. The effect of IVF on the health of newborn babies is still controversial, which was discussed in a review and focused on the issues about the neuro-psycho-motor area [30].

Gene therapies for infertility are a rising topic, but autoimmune disorders like thyroid autoimmunity make the treatment difficult. This condition is a cause of infertility, miscarriage, and preterm delivery. A paper showed that thyroid autoimmunity may be curable with Mesenchymal Stem Cells, which could be an essential step to prevent infertility caused by thyroid autoimmunity [31].

Another point worth discussing is the psychological effect of IVF treatment on couples receiving ART. The well-being of infertile couples is a medical problem that affects the treatment outcome, as anxiety, depression, and feelings of helplessness cause noncompliance with the treatment. Research revealed that focusing on the evolutionary implications of parental competence on the development of children is significant [32].

Our research had many strengths, which in our opinion makes it a valuable contribution to the literature. First, the association between TSH and AMH was analyzed in a polynomial manner, which enabled us to find a value of TSH that yields the highest AMH levels. This is a novel finding in itself and the researchers could not find any other study that reports an optimal TSH value. Additionally, our sample size was significantly larger than many studies done on this topic. Also, an upper or lower limit of TSH was not set while including participants, which allowed the analysis of all TSH levels, within or not within the designated healthy range. Finally, the Twang toolkit was utilized, which is a newly developed approach to weighting and analysis of nonequivalent groups [25]. With this method, the effect of age on TSH was overcome. Consequently, the association between TSH and AMH was analyzed.

Our study also had some limitations. Most importantly, this was a retrospective study, so it carries all the limitations that come along with it. Additionally, this study was performed using data from women referred to a single clinic with the complaint of infertility. Therefore, due to only utilizing data from infertile patients from a single center, the results for the general population cannot be represented within our research. In addition, our study does not have a control group, which decreases the internal validity. Due to the limitations of the Twang library, a polynomial regression model could not be utilized. Only linear regression models were utilized using age bias-free TSH data. Another limitation of our study was that thyroid autoimmunity, triiodothyronine (T3), and thyroxine (T4) levels could not be analyzed due to not having such data in our medical records. This limitation can be overcome by conducting a prospective study. Similarly, inhibin B could not be analyzed as an ovarian reserve marker. However, for ovarian reserve, AMH remains the preferred marker [33], and accordingly, the association between TSH and AMH was mainly focused on in our analyses. Finally, IVF treatment results were not considered as an outcome measure, as this was not within the purposes of this study.

Another point that we would like to discuss and think might be an important point in the association between thyroid dysfunction and ovarian reserve is the fertility status of the patients. According to the literature research we conducted, we believe that there is a chance that this parameter might be the main one affecting the presence of an association. Many studies performed on infertile populations showed an association [9–11], while those concerning fertile populations showed no association [10, 12]. Further studies with methodologies that investigate the effect of this parameter can be performed to enlighten this controversial topic. Additionally, guidelines differ in their recommendation regarding TSH testing in infertile women. Some recommend it to all while others recommend it to only symptomatic patients. A systematic review is needed to clarify this point.

## Conclusions

In conclusion, our study shows a correlation between TSH and AMH values in a population of infertile women. This correlation was best described in a 2^nd^ degree polynomial regression model, and the inflection point of the model was found to be 2.88 mIU/L. This means that the highest AMH result is yielded by a TSH value of 2.88 mIU/L, which can be interpreted as both high and low TSH levels result in low AMH levels as they deviate from the optimal TSH level, 2.88 mIU/L. It was also found that AMH and AFC were positively correlated, while AMH and FSH were negatively correlated. Further studies are needed to enlighten this controversial topic and to understand the clinical implications of our findings.

### Supplementary Information

**Additional file 1. **Appendix Table.

## Data Availability

The datasets used and analyzed during the current study are available from the corresponding author on reasonable request.

## Abbreviations

FSH: Follicle-stimulating hormone
AMH: Anti-Müllerian hormone
AFC: Antral follicle count
TSH: Thyroid-stimulating hormone
IVF: In vitro fertilization
FIGO: The International Federation of Gynecology and Obstetrics
BMI: Body mass index
ATADEK: Acıbadem Health Group Ethical Board
SPSS: Statistical Product and Service Solutions
ART: Assisted-reproductive technologies
T3: Triiodothyronine
T4: Thyroxine
