# Supplementary material for: Thyroid hormones and ovarian reserve: a comprehensive study of women seeking infertility care
Source: BMC Womens Health. 2023 Nov 4;23:570. doi: 10.1186/s12905-023-02725-1 (PMC10625203; doi:10.1186/s12905-023-02725-1)
Supplement: Supplementary file 1 — Additional file 1. Appendix Table. [file 12905_2023_2725_MOESM1_ESM.docx]

| Variables | Estimate | Standard Error | t value | p value |
| --- | --- | --- | --- | --- |
| Linear Regression Model | | | | |
| Intercept | 1.29 | 0.09 | 13.99 | <0.001 |
| TSH | -0.01 | 0.04 | -0.27 | 0.79 |
| 3^rd^ Degree Polynomial Regression Model | | | | |
| Intercept | 0.74 | 0.19 | 3.93 |  |
| 1^st^ Slope | 0.62 | 0.21 | 2.93 | 0.003 |
| 2^nd^ Slope | -0.17 | 0.07 | -2.55 | 0.011 |
| 3^rd^ Slope | 0.01 | 0.01 | 1.99 | 0.057 |
| 4^th^ Degree Polynomial Regression Model | | | | |
| Intercept | 0.79 | 0.24 | 3.25 | 0.001 |
| 1^st^ Slope | 0.51 | 0.38 | 1.34 | 0.181 |
| 2^nd^ Slope | -0.11 | 0.19 | -0.57 | 0.571 |
| 3^rd^ Slope | -0.001 | 0.03 | -0.03 | 0.974 |
| 4^th^ Slope | <0.001 | 0.001 | 0.33 | 0.739 |
| 10^th^ Degree Polynomial Regression Model | | | | |
| Intercept | 1.73 | 0.52 | 3.33 | <0.001 |
| 1^st^ Slope | -5.05 | 3.98 | -1.27 | 0.204 |
| 2^nd^ Slope | 9.52 | 0.10 | 0.88 | 0.380 |
| 3^rd^ Slope | -7.38 | 0.14 | -0.53 | 0.597 |
| 4^th^ Slope | 2.76 | 9.93 | 0.28 | 0.782 |
| 5^th^ Slope | -0.41 | 4.24 | -0.10 | 0.923 |
| 6^th^ Slope | -0.04 | 1.12 | -0.04 | 0.971 |
| 7^th^ Slope | 0.03 | 0.19 | 0.14 | 0.891 |
| 8^th^ Slope | -0.004 | 0.02 | -0.21 | 0.832 |
| 9^th^ Slope | <0.001 | 0.001 | 0.27 | 0.788 |
| 10^th^ Slope | >-0.001 | <0.001 | -0.31 | 0.754 |

***Table Title:*** Appendix Table.

***Table Legend:*** Linear regression models and polynomial regression models (2^nd^, 3^rd^, 4^th^, 10^th^ degrees) with TSH data for AMH prediction. Estimates, standard errors, t values and p-values are provided.
